# Delaying rewards reduces egoism bias during prosocial acts

**DOI:** 10.1093/scan/nsag030

**Published:** 2026-05-07

**Authors:** Guanglong Liu, Wendeng Yang, Ya Zheng

**Affiliations:** Department of Psychology, Guangzhou University, Guangzhou 510006, China; Center for Reward and Social Cognition, School of Education, Guangzhou University, Guangzhou 510006, China; Department of Psychology, Guangzhou University, Guangzhou 510006, China; Center for Reward and Social Cognition, School of Education, Guangzhou University, Guangzhou 510006, China; Department of Psychology, Guangzhou University, Guangzhou 510006, China; Center for Reward and Social Cognition, School of Education, Guangzhou University, Guangzhou 510006, China

**Keywords:** egoism bias, reward evaluation, delay discounting, prosocial behavior, EEG

## Abstract

People tend to prioritize their own benefits over those of others during reward evaluation, a tendency known as egoism bias. Recent evidence suggests that delaying rewards may reduce this bias in prosocial behavior, yet the underlying mechanisms remain unclear. This study investigated this issue by recording EEG activity when participants (*N *= 40) evaluated immediate or delayed rewards for themselves and an anonymous person during a prosocial reward task. We found an egoism bias in immediate reward evaluation, with stronger preferences and neural reward sensitivity (as indexed by the reward positivity, P3, and delta power) for self-benefiting compared to other-benefiting trials. Crucially, this egoism bias decreases when rewards were delayed, as shown by comparable reward sensitivity, overlapping neural representations, and positive neural correlations between self-benefiting and other-benefiting trials. These convergent findings establish reward deferral as a critical variable in reducing egoism bias during prosocial decision-making.

## Introduction

When evaluating outcomes that benefit oneself versus others, do people tend to favor their own interests? While studies demonstrate that vicarious reward processing influences prosocial behavior (Ruff and Fehr 2014), evidence indicates that people consistently value rewards less for others than for themselves (Jones and Rachlin 2006), show less emotional engagement in others’ gains (Albrecht *et al*. 2011), and put less effort into the actions benefiting others (Lockwood *et al*. 2017). This self-focused tendency in reward-related processing, hereafter referred to as egoism bias, reflects a robust self-other asymmetry in reward evaluation. Although this asymmetry may arise from multiple factors, one important issue is whether and how it can be reduced to promote prosociality (Pronin *et al*. 2008, Varnum *et al*. 2014).

Emerging evidence shows that delaying rewards may reduce the egoism bias in prosocial behavior. According to construal level theory (Trope and Liberman 2003), people think about future events more vaguely and abstractly, while imaging nearer events in vivid, concrete details. As two forms of psychological distance, social and temporal distance share common psychological and neural correlates, as revealed by the delay discounting task and the social discounting task. Both delay and social discounting are associated with the “extension of the self” (Rachlin and Jones 2010), follow a roughly similar hyperboloid pattern (Rachlin and Jones 2008), and are similarly responsive to decision policies (Molouki and Bartels 2020). Moreover, these two forms of psychological distance are related such that the more people discounted monetary rewards for themselves in the future, the more they discounted monetary rewards for socially distant others (Rachlin and Jones 2008, Story *et al*. 2020). Neuroimaging studies found that overlapping neural circuits are engaged in both delay discounting and social discounting, including the dorsomedial prefrontal cortex (Piva *et al*. 2019), the temporo-parietal junction (Soutschek *et al*. 2016), and the mesolimbic reward networks (Hill *et al*. 2017).

Based on shared functional correlates between delay and social discounting, some researchers have proposed that similar interventions may be successful for both types of discounting. On the one hand, some studies using delay discounting tasks demonstrate that people are more impulsive when waiting for personal rewards but more patient when rewards benefit others (Ziegler and Tunney 2012, Charlton *et al*. 2013). On the other hand, other studies using social discounting tasks demonstrate that postponing a reward for oneself and for another person by an equal amount of time reduces the egoism bias in social discounting, showing as a pattern of future altruism (Yi *et al*. 2011, Osinski and Karbowski 2017). Despite these findings, the mechanisms by which time delay reduces egoism bias remain unclear. Specifically, previous behavioral discounting tasks derive the subjective value of delayed or others’ rewards as a relative value, with immediate or self’s rewards serving as the reference (Kirby *et al*. 1999). This leaves open the question of whether future altruism observed in prior research is driven by hyposensitivity to immediate rewards, hypersensitivity to delayed rewards, or both. These possibilities could be tested by measuring neural responses during prosocial behavior as people evaluate immediate and delayed rewards for themselves and others.

In this study, we recorded EEG activity when participants performed a prosocial reward task to earn monetary rewards for themselves or an anonymous other person. They experienced four types of rewards as a function of reward magnitude (low vs high) and reward time (immediately vs 6 months later) separately for themselves and others. We focused on the reward positivity (RewP) and P3 of the event-related potential (ERP) component, which have been established as neural signatures of reward evaluation, with its amplitudes being larger for self’s rewards compared to other’s rewards (Leng and Zhou 2010, Ma *et al*. 2011, Xu 2021, Zhang *et al*. 2023). We predicted that effect of reward magnitude on the RewP and P3 would be greater in self-benefiting trial than other-benefiting trials, showing as an egoism bias. We hypothesized that this egoism bias would be reduced as the delay to receive a reward increases. Specifically, reward effects would be comparable between self-benefiting versus other-benefiting trials when rewards are delivered in the future. Moreover, we explored theta and delta oscillation, two time-frequency signatures of reward evaluation (Glazer *et al*. 2018), to provide more evidence for our hypothesis.

## Materials and methods

### Participants

We recruited 42 right-handed young adults for this study. Two were excluded from data analysis due to their insufficient artifact-free trials (less than 50%) in the EEG experiment. The final sample consisted of 40 participants (20 females; *M *= 19.60 years, *SD *= 1.17). Our sample size was determined based on previous ERP studies examining reward processing in self-other contexts (e.g. Xu 2021, Zhang *et al*. 2023). To evaluate statistical power, we conducted a sensitivity analysis using the *simr* v.1.0.7 package (Green and MacLeod 2016), comparing the regression weight (*β*) for each effect of interest with the smallest detectable effect size at 80% power given the current sample. Results indicated that most of the significant effects observed the threshold, suggesting that the study was adequately powered with 40 participants (see Supplementary Table 1 for detailed results). All participants had normal or corrected-to-normal vision and reported no psychiatric or neurological conditions. Each participant received a base payment of ¥15, a bonus of ¥22 earned personally, and an additional bonus of ¥22 earned by another anonymous person (see details in *Procedure*). The research protocol was approved by a local institutional review board in Guangzhou University, and each participant provided written informed consent before the experiment.

### Procedure

We used a prosocial reward task adapted from a previous study (Zheng, Guan, *et al*. 2023) and manipulated three within-subject factors using a 2 (beneficiary: self, other) × 2 (magnitude: small, large) × 2 (time: immediate, delayed) factorial design. In this task, participants played a simple guessing game to earn rewards for themselves (self-benefiting trials) and the next participant (other-benefiting trials). The rewards were low or high in magnitude and immediate or delayed in time. Before the task, each participant received an envelope containing rewards earned for them by the previous participant, but the exact amount remained unclosed. Following task instructions, participants were comfortably seated 60 cm away from a computer screen in a dimly lit, sound attenuating chamber. After EEG setup, they performed the task that was administered using E-prime 2.0 software (Psychology Software Tools, USA). To minimize the influence of social norms such as reciprocity (Gintis *et al*. 2003), participants never met in person and were assured of their anonymity.

**Figure 1:**
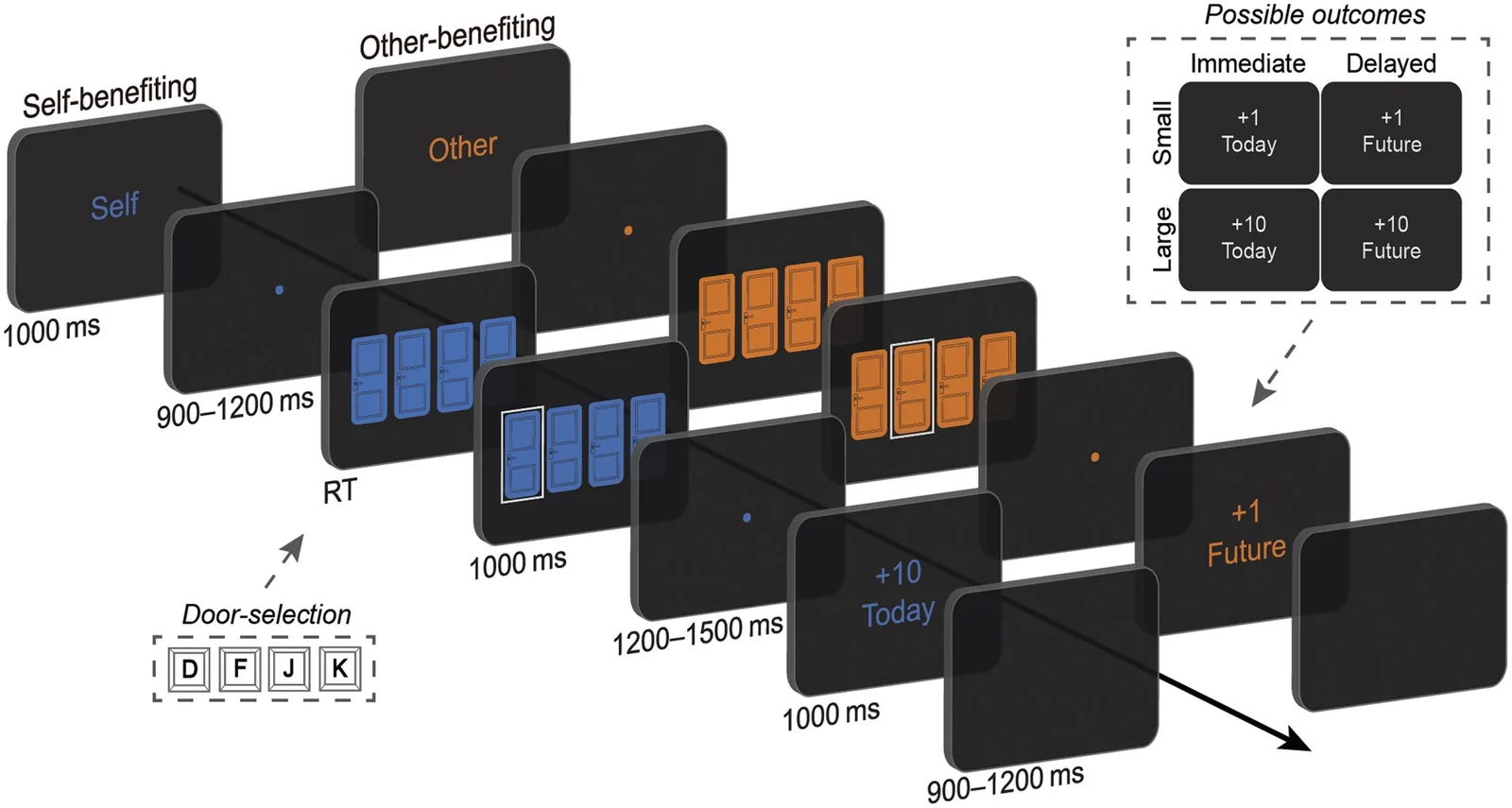
Schematic representation of the prosocial reward task. In each trial, participants selected one of four doors either for themselves or for others and received feedback showing whether their choice yielded a small or large reward, delivered immediately or 6 months later. RT, reaction time.

As illustrated in Fig. 1, each trial started with a colored cue (blue or orange) at the center of the screen for 1000 ms, indicating whether the beneficiary was the participants themselves or the next participant. The color-to-beneficiary mapping was counterbalanced across participants and remained constant throughout the trial as a reminder. After a jittered interval of 900–1200 ms, four doors appeared in a horizontal line. Participants were required to select one door by pressing a corresponding button with their left or right index or ring fingers to earn monetary rewards. Each door contained one of four possible outcomes: ¥1 or ¥10, delivered either immediately after the experiment or 6 months later. Upon their response, the chosen door was highlighted with a white box for 1000 ms. After another jittered interval of 1200–1500 ms, the outcome was displayed for 1000 ms, showing the time and magnitude of the reward. Each of the four outcomes occurred 40 times in a pseudorandomized sequence during both self-benefiting and other-benefiting trials. Trials were interleaved with an intertrial interval varying between 900 and 1200 ms.

The task consisted of 320 trials divided into eight blocks, with brief breaks between blocks. The trials were randomly mixed between two types: self-benefiting trials, where participants earned rewards for themselves, and other-benefiting trials, where they earned rewards for the next participant. Participants were informed that four trials from each type would be randomly drawn to determine the final payment for themselves and the next participant, respectively. Unbeknownst to them, each payment was fixed as two ¥1 and two ¥10 trials, with at least one trial from each time period. Immediate rewards were delivered at the end of the experiment, while delayed rewards were transferred to participants’ bank accounts 6 months later. Participants were explicitly informed of this arrangement to ensure that the delay was perceived as a postponement of reward delivery rather than uncertainty about reward receipt. To enhance the perceived credibility of this procedure, participants recorded their delayed rewards and the corresponding delivery time, which was then sealed in an envelope and retained by the experimenter until the specified payment date. Before the experiment, participants were shown examples of completed envelopes from earlier participants to familiarize them with the documentation processes. As both the experimenters and participants were affiliated with the same university, the delay payment procedure was regarded as trustworthy. To familiarize participants with the task, eight practice trials were provided before the formal experiment. At the end of the experiment, participants rated their liking of the four outcome types for both self-benefiting and other-benefiting trials using a nine-point Likert scale (1 = “not at all”; 9 = “very much”).

### EEG recording and processing

EEG data were recorded using a 64-channel system, aligned with the 10-20 system and referenced against the left mastoid. A pair of electrodes were placed at the outer canthi of both eyes for horizontal electrooculogram (EOG) and another pair of electrodes above and below the left eye for vertical EOG. Both EEG and EOG signals were amplified with a Neuroscan SynAmps^2^ amplifier with a band-pass filter of 0.05–200 Hz and digitized at a sampling rate of 500 Hz. Electrode impedances were maintained below 5 KΩ.

EEG data were analyzed using custom MATLAB (v2020b; The MathWorks Inc) scripts and EEGLAB (v2021.0; Delorme and Makeig 2004) and ERPLAB (v8.10; Lopez-Calderon and Luck 2014) functions. All channel data were band-pass filtered between 0.1–35 Hz using a zero phase-shift Butterworth filter (12 dB/octave roll-off) and then rereferenced to the averaged mastoids. Bad channels were interpolated using the EEGLAB spherical interpolation algorithm. Portions of EEG data with extreme voltage offsets or break periods were removed through two automatic ERPLAB algorithms. Subsequently, the continuous EEG data were entered into an independent component analysis to remove ocular and muscle artifacts, aided by the ICLabel algorithm (Pion-Tonachini *et al*. 2019). Data were then segmented from 200 ms before to 1000 ms after the presentation of feedback stimuli, with the averaged prestimulus activity as a baseline. Finally, an artifact detection algorithm was used to remove epochs containing a voltage difference > 50 μV between sample points, a voltage difference > 200 μV within an epoch, a maximum voltage difference < 0.5 μV within 100 ms intervals, or a slow voltage drift with a slope > ±100 μV. An average of 98.30% artifact-free trials were retained for statistical analysis (see Supplementary Table 2 for detailed trial number). Single-trial RewP (split-half reliability *r *= 0.97) was measured as the mean activity from 260 to 360 ms post-feedback onset over frontocentral areas (Fz, FCz), and the P3 (split-half reliability *r *= 0.97) from 320 to 420 ms over centroparietal areas (CPz, Pz). Measurement parameters were determined based on grand-averaged waveforms and topographic maps across conditions (Luck and Gaspelin 2017).

We performed a time-frequency decomposition per trial in 3500-ms epochs, with 1500 ms before feedback onset and through 2000 ms after. We convolved single-trial EEG activity using a set of complex Morlet wavelets ranging from 1 to 30 Hz in 30 logarithmically spaced steps. The wavelet cycles increased from 3 to 10 in the same number of steps. We used a welding baseline method to normalize single-trial EEG power values for each frequency band. Specifically, EEG power values in the baseline interval (from -500 to -300 ms) were combined across all trials into a single, long baseline. Each trial was then *z*-scored by the average power value of this welding baseline. This method was chosen to provide a more unbiased estimate compared to other single-trial normalization methods (Ciuparu and Mureşan 2016). Using a similar orthogonal selection approach, single-trial theta power (split-half reliability *r *= 0.94) was measured as the mean activity from 150 to 450 ms over 4–7 Hz at FCz post feedback onset, and delta power (split-half reliability *r *= 0.97) from 200 to 500 ms over 1–3 Hz at CPz.

### Statistical analysis

Single-trial ERP and EEG power data were analyzed using linear mixed-effects regression models using the *lme4* package (v1.1.35; Bates *et al*. 2015) in *R* (v4.3.3). Each model included beneficiary (−0.5 for self-benefiting and +0.5 for other-benefiting), magnitude (−0.5 for small and +0.5 for large), time (−0.5 for immediate and +0.5 for delayed), and their interactions as fixed effect predictors. Random effects components included random slopes for the first-level variables and a subject-level random intercept. Models were estimated using restricted maximum likelihood. For each model, we initially fitted the maximal random-effects structure justified by the design. When the model was overparameterized, we applied singular value decomposition to iteratively simplify the random-effects structure until the model converged. *P* values were computed using the *sjplot* package (v2.8.15; Lüdecke *et al*. 2021). Significant interactions were decomposed using pairwise comparisons of estimated marginal means, as implemented in the *emmeans* package (v1.10.1; Lenth 2022), with all pairwise contrasts corrected for multiple comparisons using the false discovery rate (FDR) method. Post-experimental rating data of liking were analyzed using a repeated-measures analysis of variance (ANOVA) with beneficiary, magnitude, and time as within-subjects factors.

In addition to univariate ERP analysis, we performed multivariate representational similarity analysis (RSA) on our EEG data to examine (i) the neural representations of beneficiary, reward magnitude, and reward time and (ii) how the neural representations of beneficiary and magnitude are modulated by reward time. We constructed three model representational dissimilarity matrixes (RDMs) related to different task variables, separately for beneficiary (self vs other), magnitude (small vs large), and time (immediate vs delayed). We computed an 8 × 8 neural RDM based on the ERP topographies (60 channels) at each time point by measuring the Mahalanobis distance between conditions (Hall-McMaster *et al*. 2019). We then converted the upper triangular portion of each matrix into a vector, *z*-scored both model and neural RDMs, and conducted a multiple regression analysis at each time point for each participant. This yielded a time series of regression coefficient estimates for the neural coding of beneficiary, magnitude, and time. To examine the modulation of beneficiary and magnitude coding by time, we repeated the above analyses separately for immediate and delayed reward trials. Finally, we applied cluster-based permutation testing on the time series to correct for multiple comparisons (Maris and Oostenveld 2007).

## Results

All data and code used for this study are available on OSF at https://osf.io/gn2kj/? view_only=bdf50c8c97ea469fbc6ca90010cf3c07.

### Behavioral and rating data

**Figure 2:**
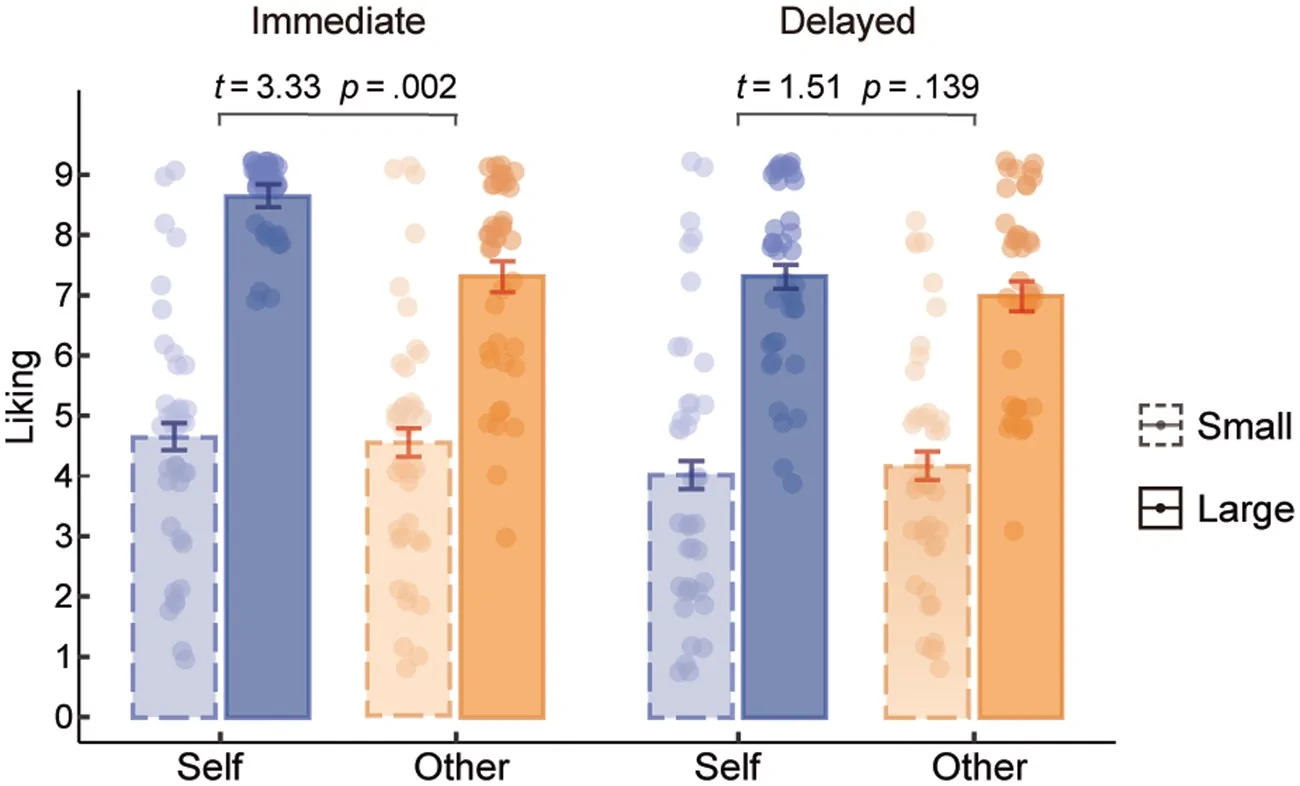
Post-experimental liking ratings. Error bars represent the within-subject standard error of the mean.

Although decision time was slower for other-benefiting trials (951 ± 848 ms) than for self-benefiting trials (945 ± 850 ms), this difference was not statistically significant (*t_39_* = −0.54, *p* = .595, Cohen’s *d *= −0.08). For liking rating data, participants preferred large rewards over small rewards (*F*(1, 39) = 143.76, *p* < .001, η_p_^2^ = 0.79) and immediate rewards over delayed rewards (*F*(1, 39) = 22.61, *p* < .001, η_p_^2^ = 0.37). We found significant two-way interactions between beneficiary and magnitude (*F*(1, 39) = 7.46, *p* = .009, η_p_^2^ = 0.16) and between beneficiary and time (*F*(1, 39) = 9.54, *p* = .004, η_p_^2^ = 0.20). These interactions were further qualified by a significant three-way interaction among beneficiary, magnitude, and time (*F*(1, 39) = 6.51, *p* = .015, η_p_^2^ = 0.14). To decompose this three-way interaction, we calculated difference scores between large and small rewards for liking ratings. Post hoc comparisons (Fig. 2) revealed that reward magnitude had a stronger effect (i.e. greater difference scores) on liking ratings in self-benefiting trials compared to other-benefiting trials for immediate rewards (*t_39_* = 3.33, *p* = .002, Cohen’s *d *= 0.59) but not for delayed rewards (*t_39_* = 1.51, *p* = .139, Cohen’s *d *= 0.23). Detailed ANOVA results are provided in Supplementary Table 3.

### EEG data

**Figure 3:**
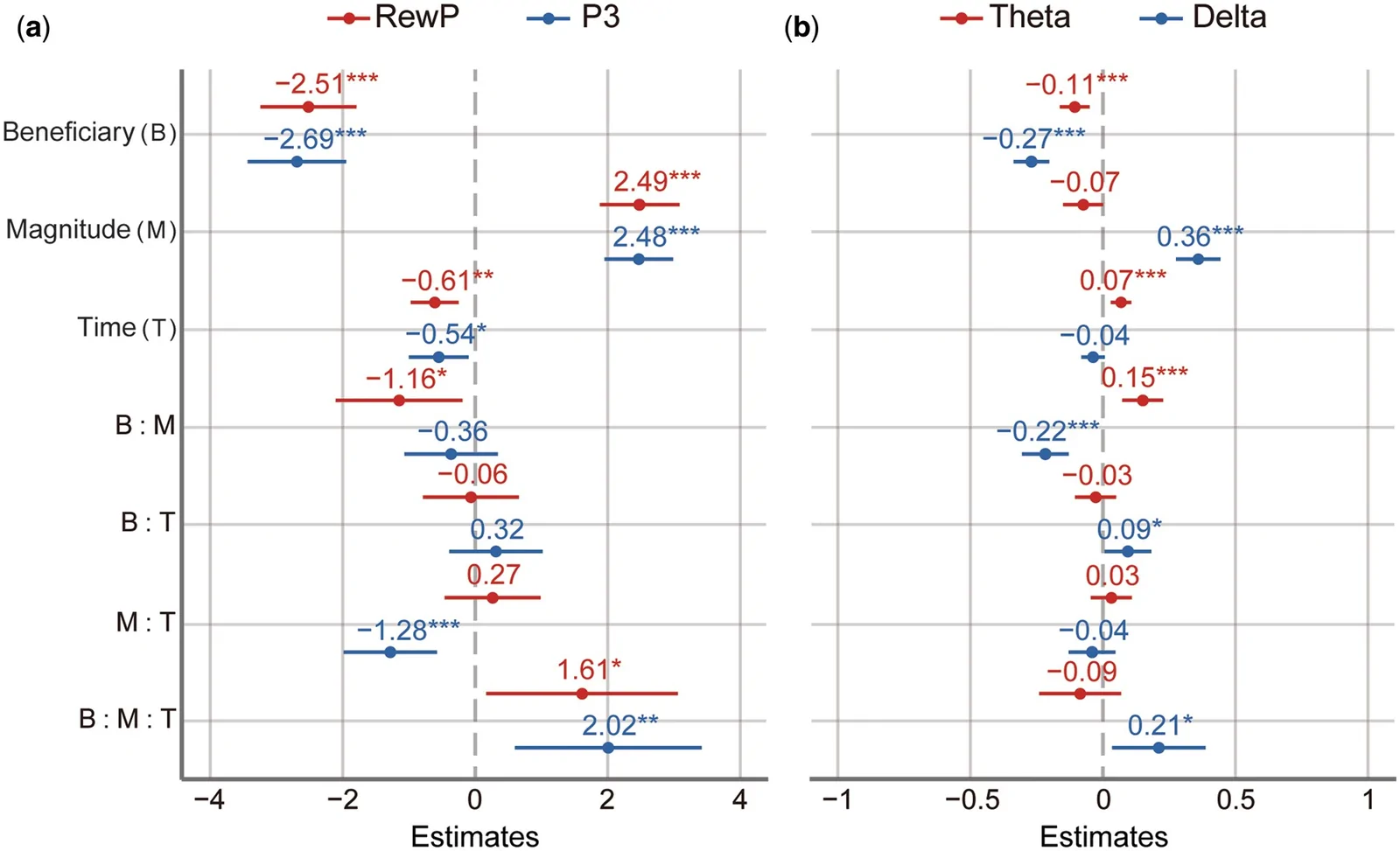
Coefficient estimates of mixed-effects models for time-domain (a) and time-frequency domain (b) data. *p <.05, **p <.01, ***p <.001.

**Figure 4:**
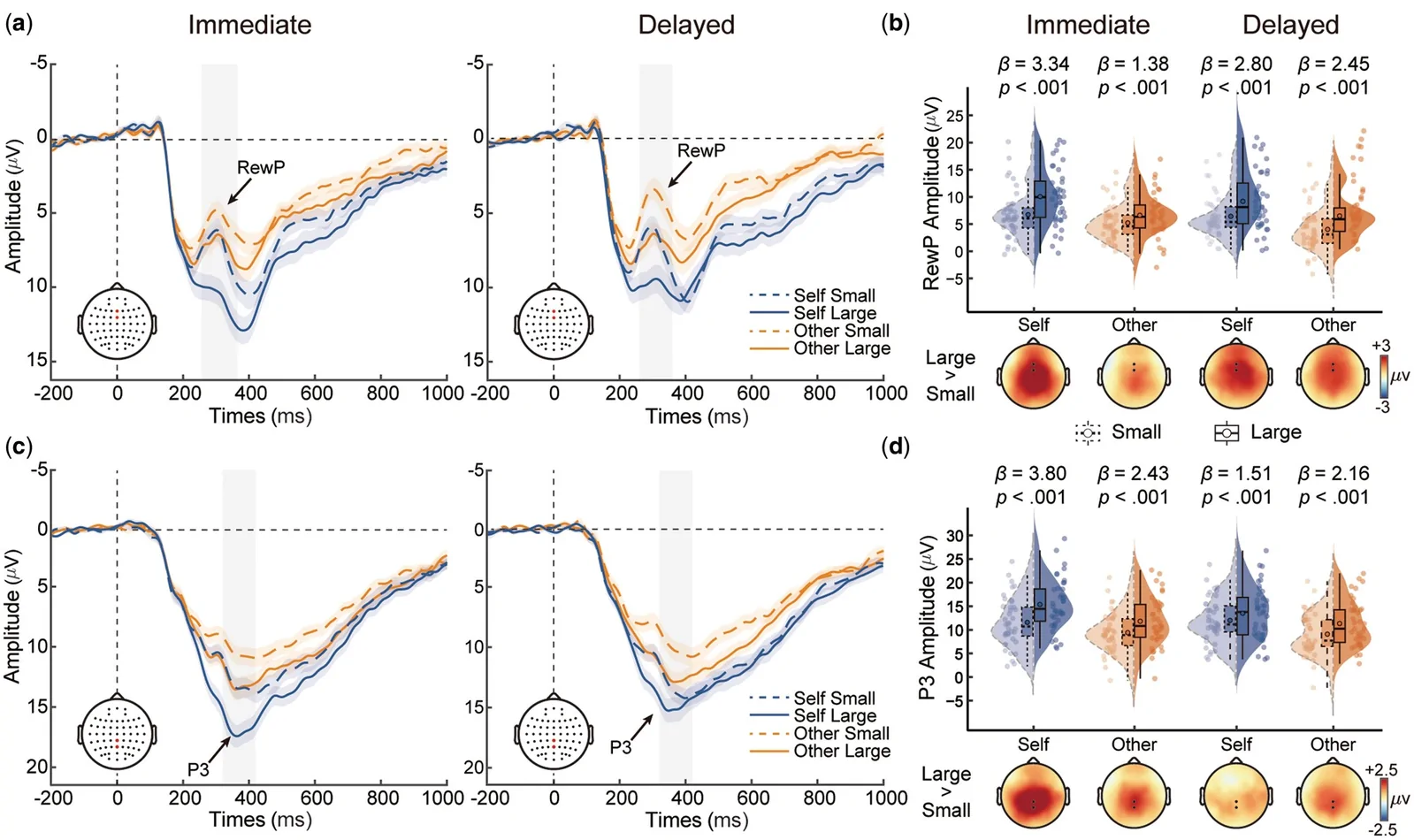
Task effects on ERP data. (a) Grand-averaged ERP waveforms over frontocentral areas. (b) Raincloud plots of the mean amplitudes for the RewP data. (c–d) same as (a–b), except that ERP waveforms represent an average over centroparietal areas, and data points represent the P3 data. For ERP waveforms, colored shaded error bars indicate standard error of the mean across participants, and gray shaded vertical bars represent time windows for quantification. For raincloud plot, the density plots depict the distributions, the boxplots represent the median and the first and third quartiles, and the colored circles and dots indicate the mean for each participant and across participants, respectively. Scalp voltage maps represent the magnitude effect (large minus small).

Figure 3 shows the estimated coefficients from the mixed-effects models for EEG data. Detailed statistical results of these mixed-effects models are shown in Supplementary Tables 4 and 5. Figure 4 illustrates the grand-averaged ERP waveforms as a function of beneficiary and magnitude separately for immediate and delayed trials. Both the RewP and P3 were significantly more positive for self-benefiting trials than for other-benefiting trials (RewP: *β* = −2.51, *t *= −6.80, *p* < .001; P3: *β* = −2.69, *t *= −7.08, *p* < .001) and for large rewards relative to small rewards (RewP: *β* = 2.49, *t *= 8.11, *p* < .001; P3: *β* = 2.48, *t *= 9.37, *p* < .001). Moreover, they were less positive for delayed rewards compared to immediate rewards (RewP: *β* = −0.61, *t *= −3.28, *p* = .001; P3: *β* = −0.54, *t *= −2.36, *p* = .023), indicating a neural manifestation of delay discounting. These results suggest that neural dynamics underlying reward evaluation are affected by the three within-subject variables of beneficiary, magnitude, and time.

#### Reward effects on the RewP and P3 are modulated by beneficiary for immediate but not for delayed rewards

While the RewP (Fig. 4a and b) tracked a significant interaction between beneficiary and magnitude (*β* = −1.16, *t *= −2.37, *p* = .023), the P3 (Fig. 4c and d) tracked a significant interaction between magnitude and time (*β* = −1.28, *t *= −3.55, *p* < .001). Importantly, both ERP components exhibited a significant three-way interaction among beneficiary, magnitude, and time (RewP: *β *= 1.61, *t *= 2.18, *p* = .029; P3: *β* = 2.02, *t *= 2.80, *p* = .005). Pairwise interaction contrasts revealed that when rewards were delivered immediately, the magnitude effect on the RewP and P3 was more pronounced for self-benefiting trials (RewP: *β* = 3.34, *z *= 6.35, *p* < .001; P3: *β* = 3.80, *z *= 9.30, *p* < .001) than other-benefiting trials (RewP: *β* = 1.38, *z *= 3.36, *p* < .001; P3: *β* = 2.43, *z *= 5.96, *p* < .001), yielding a significant interaction between beneficiary and magnitude (RewP: *β* = −1.95, *t *= −3.78, *p* < .001; P3: *β* = −1.36, *t *= −2.13, *p* = .039). In contrast, when rewards were delivered 6 months later, the magnitude effect on the RewP and P3 was comparable for self-benefiting trials (RewP: *β* = 2.80, *z *= 5.33, *p* < .001; P3: *β* = 1.51, *z *= 3.71, *p* < .001) and other-benefiting trials (RewP: *β* = 2.45, *z *= 5.97, *p* < .001; P3: *β* = 2.16, *z *= 5.29, *p* < .001), as revealed by a nonsignificant beneficiary-by-magnitude interaction (RewP: *β* = −0.34, *t *= −0.53, *p* = .597; P3: *β* = 0.65, *t *= 1.29, *p* = .199). Decomposing the three-way interaction by beneficiary revealed that, for the RewP, temporal delay did not significantly alter the magnitude effect in self-benefiting trials (*β* = 0.54, *z *= 1.03, *p* = .304) but significantly increased it for other-benefiting trials (*β* = −1.07, *z *= −2.05, *p* = .040). For the P3, temporal delay significantly the magnitude effect for self-benefiting trials (*β* = 2.28, *z *= 4.50, *p* < .001) with no significant modulation for other-benefiting trials (*β* = 0.27, *z *= 0.53, *p* = .597). These findings suggest that the reduced egoism bias for delayed rewards reflects both decreased reward sensitivity for self as indexed by the P3 and increased reward sensitivity for other as indexed by the RewP.

#### Neural coding of beneficiary is present for immediate but not for delayed rewards

**Figure 5:**
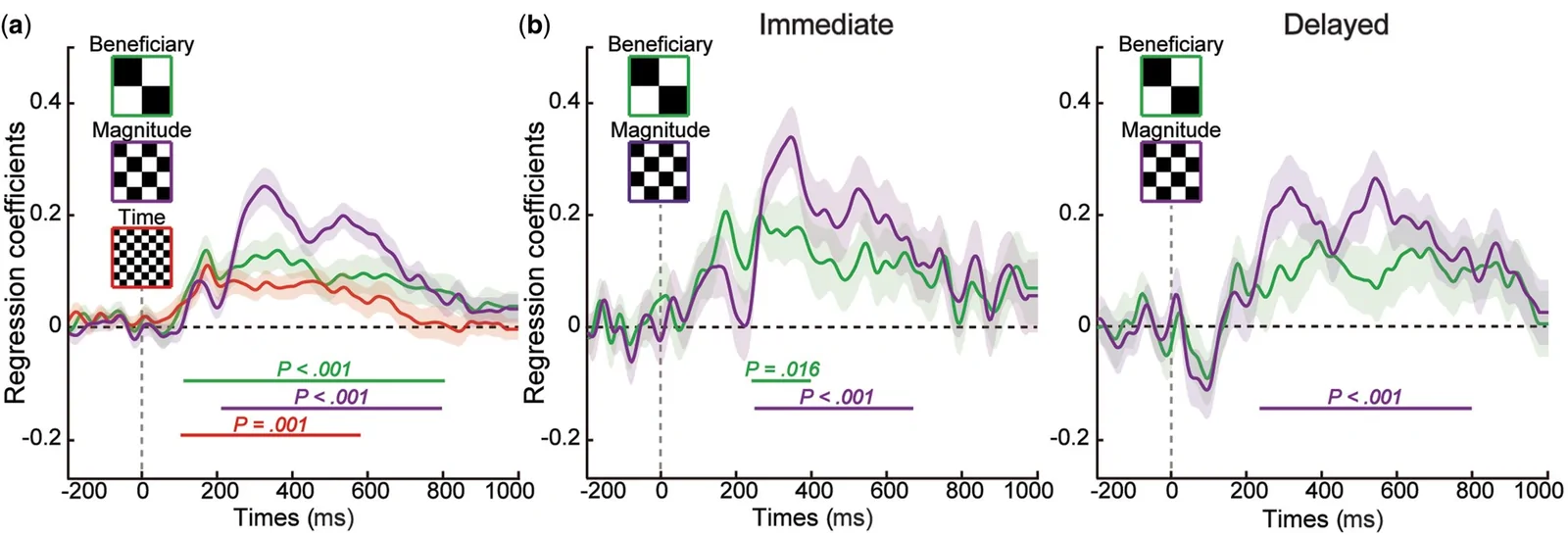
RSA results. (a) Time-resolved regression coefficients for beneficiary, magnitude, and time coding. (b) Time-resolved regression coefficients for beneficiary and magnitude coding separately for immediate and delayed rewards. Model RDMs are displayed in the top left corner. Colored shaded error bars around lines indicate standard error of the mean across participants. Colored marker lines on bottom represent significant differences from zero (cluster-corrected *p* <.05).

Next, we used multivariate RSA to examine time-resolved neural representations of beneficiary, magnitude, and time, as well as how neural representations of beneficiary and magnitude change between immediate and delayed rewards. As shown in Fig. 5a, time coding emerged first after feedback onset (103–581 ms, *p* = .001), followed by neural coding of beneficiary (111–805 ms, *p* < .001) and magnitude (211–797 ms, *p* < .001). Importantly, reward time significantly modulated the neural representations of magnitude and beneficiary. As illustrated in Fig. 5b, when rewards were delivered immediately, beneficiary coding emerged shortly after feedback onset (239–397 ms, *p* = .016), followed by magnitude coding (247–669 ms, *p* < .001). In contrast, when reward delivery was delayed, magnitude coding emerged after feedback onset (235–797 ms, *p* < .001). No reliable neural representation of beneficiary was observed. These findings suggest distinct patterns of neural coding between immediate and delayed rewards: while magnitude is coded regardless of reward time, beneficiary coding appears for immediate rewards but not for delayed rewards.

#### Reward effect on Delta power is modulated by beneficiary for immediate but not for delayed rewards

**Figure 6:**
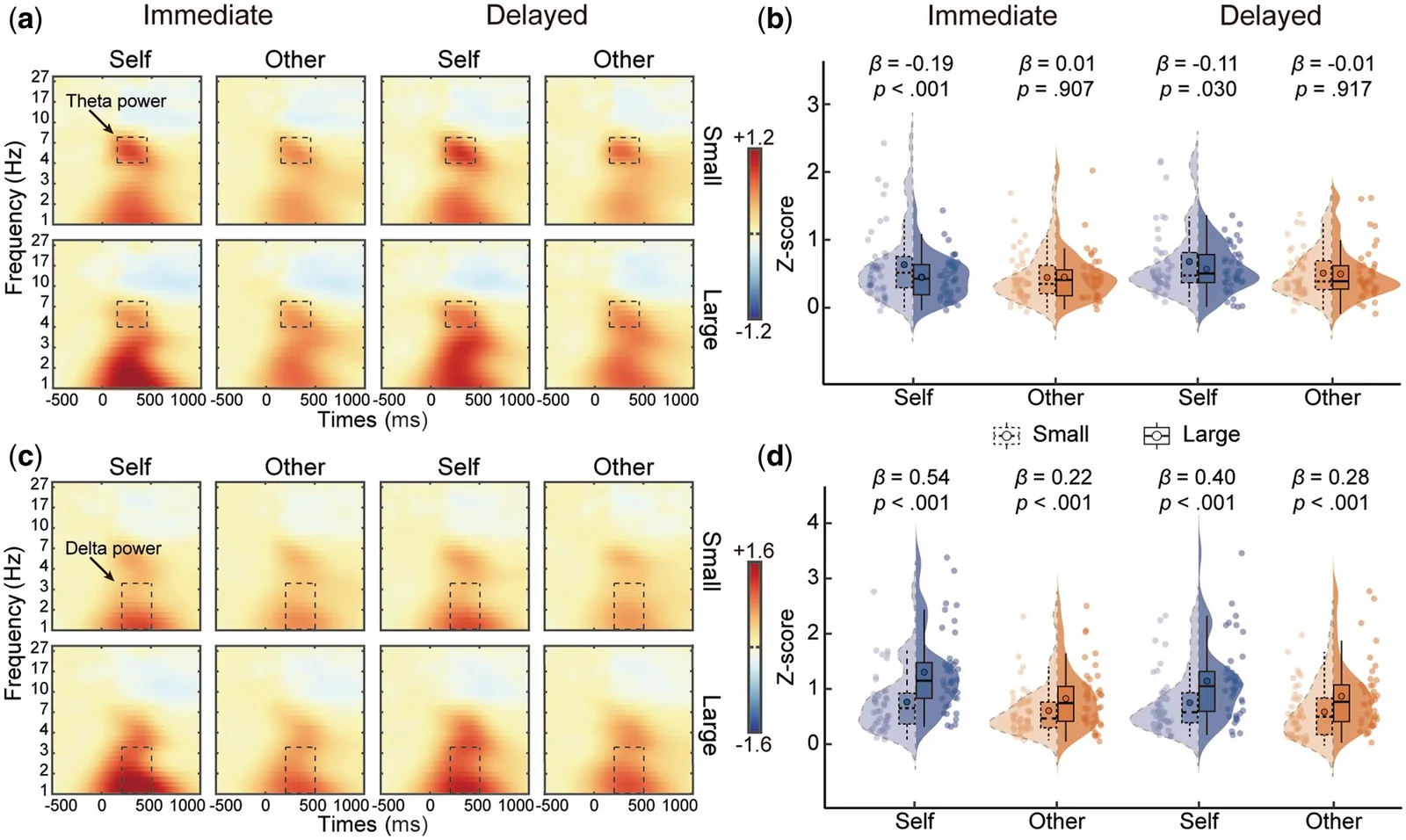
Task effects on EEG power data. Time-frequency representation and raincloud plots of theta (a–b) and delta (c–d) power data in response to small and large rewards in self- and other-benefiting trials as a function of reward time. The black boxes depict time-frequency windows used for quantification. The density plots depict the distributions, the boxplots represent the median and the first and third quartiles, and the colored dots and the black circles indicate the mean for each participant and across participants, respectively.

Figure 6 illustrates the time-frequency representations of EEG power as a function of beneficiary and magnitude separately for immediate and delayed trials. Theta power (Fig. 6a and b) was enhanced for self-benefiting compared to other-benefiting trials (*β* = −0.11, *t *= −3.73, *p* < .001) and for delayed compared to immediate rewards (*β* = 0.07, *t *= 2.77, *p* = .008). A significant interaction emerged between beneficiary and magnitude (*β* = 0.15, *t *= 3.79, *p* < .001). Post hoc comparisons revealed that for self-benefiting trials, theta power was increased for small compared to large rewards (*β* = −0.15, *z *= −3.43, *p* < .001). In contrast, for other-benefiting trials, theta power was comparable between large and small rewards (*β* < 0.01, *z *= 0.01, *p* = .994). No other significant effects were found.

Delta power (Fig. 6c and d) was increased for self-benefiting compared to other-benefiting trials (*β* = −0.27, *t *= −7.91, *p* < .001) and for large relative to small rewards (*β* = 0.36, *t *= 8.44, *p* < .001). The magnitude effect was more pronounced for self-benefiting trials (*β* = 0.47, *z *= 9.73, *p* < .001) than for other-benefiting trials (*β* = 0.25, *z *= 5.21, *p* < .001), as revealed by a significant interaction between beneficiary and magnitude (*β* = −0.22, *t *= −4.85, *p* < .001). Importantly, a significant three-way interaction among beneficiary, magnitude, and time was observed (*β* = 0.21, *t *= 2.35, *p* = .019). Pairwise interaction contrasts revealed that when rewards were immediate, the magnitude effect was more pronounced for self-benefiting (*β* = 0.54, *z *= 9.40, *p* < .001) than other-benefiting trials (*β* = 0.22, *z *= 3.80, *p* < .001), resulting in a significant interaction between beneficiary and magnitude (*β* = −0.32, *t *= −5.13, *p* < .001). In contrast, when rewards were delayed, the magnitude effect was comparable for self-benefiting trials (*β* = 0.40, *z *= 6.86, *p* < .001) and other-benefiting trials (*β* = 0.28, *z *= 4.91, *p* < .001), as revealed by a nonsignificant beneficiary-by-magnitude interaction (*β* = −0.11, *t *= −1.73, *p* = .083). Decomposing the three-way interaction by beneficiary revealed that temporal delay significantly reduced the magnitude effect for self-benefiting trials (*β* = 0.15, *z *= 2.31, *p* = .021), with no significant modulation for other-benefiting trials (*β* = −0.06, *z *= −1.01, *p* = .313). These findings further support that the reduced egoism bias for delayed rewards in delta power is driven by a self-related decrease in reward sensitivity.

#### Reward effects on the P3 and Delta power are correlated between self and others for delayed but not for immediate rewards

**Figure 7:**
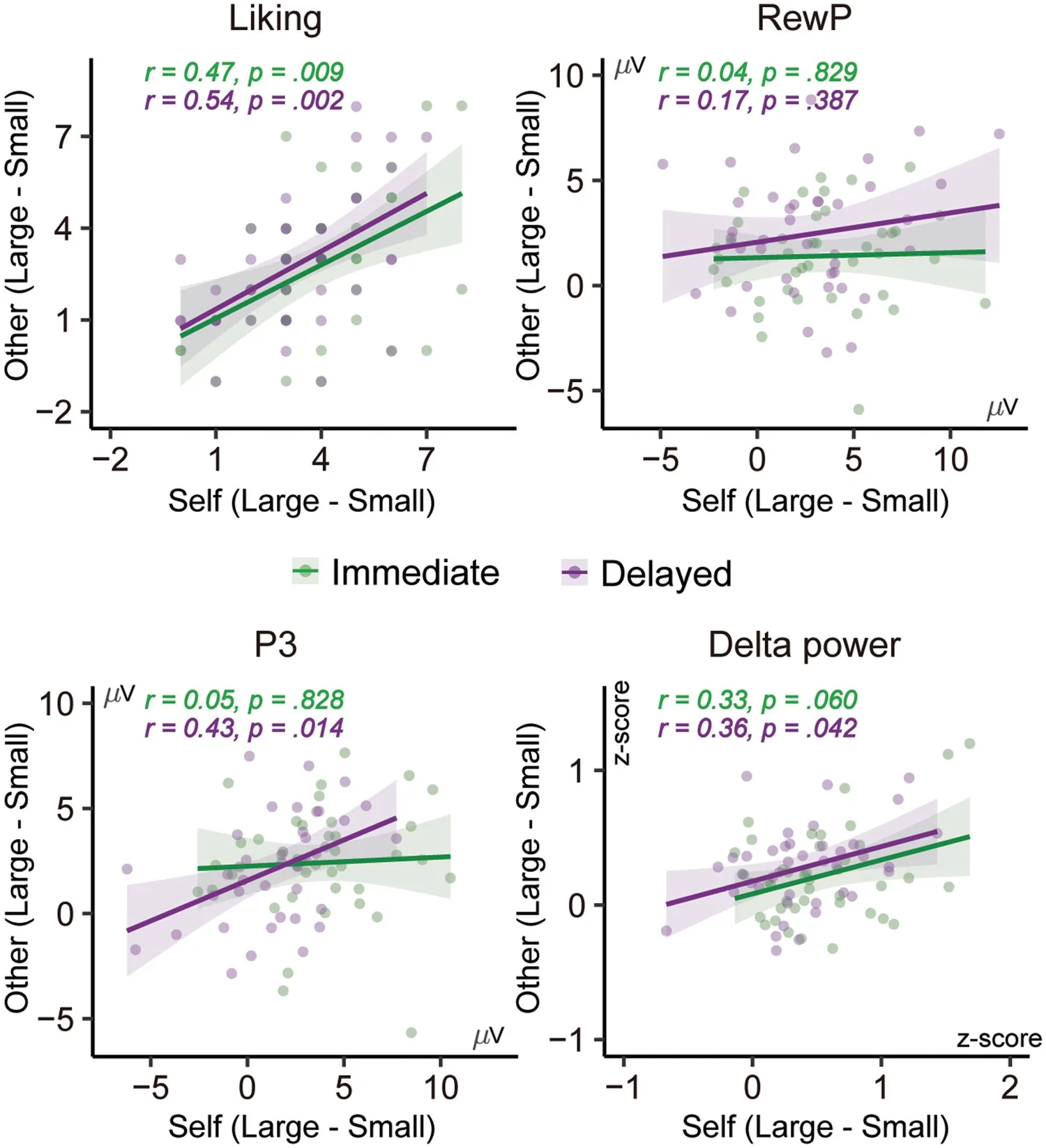
Correlations of magnitude effect (large versus small) between self-benefiting and other-benefiting trials for immediate and delayed rewards on data of self-reported liking, the RewP, P3, and delta power. All *p*-values were corrected for multiple comparisons using the false discovery rate method.

Finally, we applied Pearson’s correlation to examine the relationship of reward effect (large minus small rewards) between self-benefiting and other-benefiting trials separately for immediate and delayed rewards. Our analysis focused on the RewP, P3, and delta power, along with self-reported liking, because of the observed three-way interactions in these data. The *p* values were adjusted for multiple comparisons with an FDR of .05. As shown in Fig. 7, delayed rewards revealed significant positive correlations in both the P3 and delta power between self-benefiting and other-benefiting trials. In contrast, no significant neural correlations were found for immediate rewards. Additionally, self-reported liking showed positive correlations between self-benefiting and other-benefiting trials across both the immediate and delayed conditions. However, the correlation coefficients were not significantly different between immediate and delayed rewards (RewP: *z *= −0.65, *p* = .516; P3: *z *= −1.86, *p* = .064; Delta: *z *= −0.18, *p* = .859; Liking: *z *= −0.56, *p* = .575). These results indicate that reward effect in the delayed condition may share overlapping neural correlates between self-benefiting and other-benefiting trials.

## Discussion

In this study, we examined how delaying rewards affects egoism bias in reward evaluation. Participants performed a prosocial reward task to earn small or large monetary rewards for themselves or an anonymous person. When rewards were delivered immediately after the task, participants exhibited an egoism bias characterized by larger self-reported preferences and neural responses (the RewP, P3, and delta power) for large versus small rewards in self-benefiting trials than other-benefiting trials. In contrast, when rewards were delivered 6 months later, this egoism bias was totally inhibited such that self-reported preferences and neural responses were comparable between self-benefiting and other-benefiting trials. This future altruism was further supported by similar neural representations of beneficiary and positive neural correlations of reward effects on the P3 and delta between self-benefiting and other-benefiting trials in the delayed condition, but not in the immediate condition. Together, our findings demonstrate that delaying rewards reduces egoism bias in reward evaluation, supporting a temporal modulation hypothesis of prosocial behavior.

As expected, our ERP results revealed both the RewP and P3 were enhanced for large rewards than for small rewards, indicating a widespread reward effect on human neural circuits (Yeung and Sanfey 2004, Goyer *et al*. 2008, Zheng *et al*. 2017). These neural signatures were reduced for delayed rewards compared to immediate rewards, showing a neural discounting effect during reward evaluation (Cherniawsky and Holroyd 2013, Schmidt *et al*. 2017, Zheng, Guan, *et al*. 2023, Zheng, Shi, *et al*. 2023). Consistent with previous finding of egoism bias in reward evaluation (Leng and Zhou 2010, Ma *et al*. 2011, Xu 2021, Zhang *et al*. 2023), reward effect was smaller for other-benefiting compared to self-benefiting trials. Notably, this egoism bias has typically been observed in experimental contexts where rewards are delivered immediately after the experiment. Our findings extend these studies by incorporating a time dimension and demonstrating that reward effects on both the RewP and P3 were comparable between self-benefiting and other-benefiting trials when rewards were delivered with a time delay. These results suggest that the reduced egoism bias (i.e. future altruism) in reward evaluation may be linked to the binary evaluation as indexed by the RewP (Hajcak *et al*. 2006) and motivational salience as indexed by the P3 (Nieuwenhuis *et al*. 2005).

Our observation of future altruism was further corroborated by delta oscillation such that the stronger reward effect on delta power for self-benefiting versus other-benefiting trials during the immediate condition was reduced for the delayed condition. This finding is unsurprising, given the commonality between the delta power and P3 component (Başar *et al*. 1984), as well as the RewP (Bernat *et al*. 2015). Because delta oscillation has long been linked to affective and motivational salience (Knyazev 2007, Knyazev 2012, Glazer *et al*. 2018), our finding of the reduced egoism bias in the delayed condition may reflect that the motivation salience towards self is normalized to a baseline level when shifting from immediate to future, thus showing commonality as others. Unlike the delta power, theta power was not modulated by the three-way interaction among beneficiary, magnitude, and time, suggesting that theta-related processes are less sensitive to the temporal modulation of egoism bias. When feedback stimulus conveys multiple pieces of information, theta power responds mainly to primary feedback attributes (e.g. reward magnitude), while delta power processes both primary and higher-level secondary (e.g. beneficiary and time) attributes (Bernat *et al*. 2015). Therefore, our dissociable findings of delta and theta power suggest that future altruism in prosocial acts may be driven by later and more elaborated cognitive processes that are selectively captured by delta power rather than theta power.

Our results suggest that reduced egoism bias for delayed rewards is driven by a dual mechanism operating at distinct processing stages of reward evaluation. This provides insight into the neural basis of future altruism observed in previous studies using discounting tasks. In these tasks, participants typically choose between two options: a small immediate reward and a large future reward for themselves or others, or between a small reward for themselves and a large reward for others at varying time delays. Research consistently shows that adding temporal and social distance to the receipt of outcomes decreases social discounting (Yi *et al*. 2011, Osinski and Karbowski 2017) or time discounting (Ziegler and Tunney 2012, Charlton *et al*. 2013), respectively. Relatedly, egoism bias has been linked to impulsivity-related immediate gratification (Albrecht *et al*. 2011, Dürschmid *et al*. 2021), and delayed rewards appears to help people overcome their self-orientation bias to restore self-control (Soutschek *et al*. 2016). While these findings indicate an interaction between time and social distance, the underlying mechanism has remained unclear. For example, decreased social discounting over longer delays could stem from hyposensitivity to immediate rewards, hypersensitivity to delayed rewards, or a combination of both. Our finding revealed that, at later evaluative stage as indexed by the P3 and delta power, temporal delay reduced the magnitude effect selectively for self-benefiting trials, whereas at the earlier evaluative stage as indexed by the RewP, temporal delay enhanced the magnitude effect selectively for other-benefiting trials. These results are consistent with the construal level theory (Trope and Liberman 2010), which posits that people typically form more abstract mental representations (i.e. higher level construals) of distant-future events than near-future events. While high-level construals are abstract mental representations that capture the essential, core features of future events, low-level construals focus on specific, contextual details (Trope and Liberman 2003). Under such abstract construal, the concrete self-prioritization that typically dominates immediate reward evaluation may be attenuated, reducing the motivational salience of self-rewards as reflected in the P3 and delta power. Meanwhile, the weakened self-prioritization may also release early evaluative resources from self-relevant dominance, allowing more differentiated reward appraisal for others as reflected in the RewP.

Our RSA results provided further support for the inhibited egoism bias in delayed reward context. During the immediate condition, we observed a reliable neural representation of beneficiary across the RewP and P3 periods (239–397 ms post-feedback). However, this neural representation disappeared when reward delivery was delayed. The absence of beneficiary coding for delayed rewards possibly reflects an overlapping neural representation of self and other where the perception of the two is merged (Aron *et al*. 1991, Paladino *et al*. 2010). This possibility is further strengthened by our correlational results. Specifically, when rewards were delayed, reward effects on both the P3 and delta power were positively correlated between self-benefiting and other-benefiting trials. In contrast, this correlation vanished during immediate reward delivery. Recent research has demonstrated that such overlapping self-other representations are associated with social closeness and prosocial behavior, including a greater willingness to exert cognitive effort for others (Depow *et al*. 2022), to allocate money to others (Feng *et al*. 2020), and to donate an organ to a stranger (Brethel-Haurwitz *et al*. 2018). Our findings extend these studies by showing that temporal delay elicits neural overlapping between self and other during reward evaluation. Future research should examine whether this self-other overlapping can predict real-world prosocial behaviors.

There are several limitations of this study that warrant cautions interpretation of our findings. First, the present findings are based on a single, relative long delay (6 months) with an anonymous other, and therefore should not be generalized to temporal delays or social distances broadly. Recent studies using the social delay discounting paradigm have shown that the effect of delay on self-other differences as a function of both the duration of delay and the social closeness of the beneficiary (van de Groep *et al*. 2023, van Rijn *et al*. 2024). For example, self-other differences in subjective value may persist at shorter delays with unknown others (van Rijn *et al*. 2024). Further work should systematically vary both the delay duration and the social distance of recipients (Jones 2022) to identify how self-other differences in reward evaluation evolve across these dimensions, including determining the critical threshold at which reward evaluation shifts from egoism bias to more prosocial processing towards socially close others, such as family members and friends. Second, although participants were assured of delayed reward delivery, we cannot entirely rule out that subjective uncertainty associated with future events may have contributed to the reduced reward sensitivity in the delayed condition (Muir *et al*. 2021). Future studies should directly assess subjective uncertainty to disentangle its effects from objective delay. Third, our sample consisted exclusively of Chinese young adults. Given that temporal discounting and prosocial tendencies vary across developmental stages (van de Groep *et al*. 2023, van Rijn *et al*. 2024) and cultural contexts, future research should examine whether the present findings generalize to other age groups and cultural populations. Finally, it will be important to explore whether outcome delay similarly reduces egoism bias when preventing losses for others rather than obtaining gains for them (Green and Myerson 2004).

In conclusion, this study demonstrates that egoism bias in reward evaluation decreases when rewards are delayed. This future altruism was evident as comparable reward sensitivity, overlapping neural representations, and positive neural correlations between self-benefiting and other-benefiting trials when rewards shifted from immediate to delayed delivery. These convergent findings establish reward deferral as a critical variable in reducing egoism bias during prosocial acts.

## Supplementary Material

nsag030_Supplementary_Data

## Data Availability

All data and code used for this study are available on OSF at https://osf.io/gn2kj/? view_only=bdf50c8c97ea469fbc6ca90010cf3c07.
