# Supplementary material for: Delaying rewards reduces egoism bias during prosocial acts
Source: Soc Cogn Affect Neurosci. 2026 May 7;21(1):nsag030. doi: 10.1093/scan/nsag030 (PMC13221249; doi:10.1093/scan/nsag030)
Supplement: nsag030_Supplementary_Data [file nsag030_supplementary_data.doc]

**Supplementary Materials**

**Table S1. Results from the sensitivity analysis**

| Regression equation | Tested effect | *p* | Original effect  (*β* ± *SE*) | Smallest effect  (*β*) |
| --- | --- | --- | --- | --- |
| RewP ~ Beneficiary × Magnitude × Time | Beneficiary (B) | **< .001** | -2.51 ± 0.37 | -1.06 |
|  | Magnitude (M) | **< .001** | 2.49 ± 0.31 | 0.87 |
|  | Time (T) | **.001** | -0.61 ± 0.18 | -0.51 |
|  | B:M | **.023** | -1.16 ± 0.49 | -1.36 |
|  | B:T | .873 | -0.06 ± 0.37 | -1.01 |
|  | M:T | .468 | 0.27 ± 0.37 | 1.03 |
|  | B:M:T | **.029** | 1.61 ± 0.74 | 1.97 |
| P3 ~ Beneficiary × Magnitude × Time | Beneficiary (B) | **< .001** | -2.69 ± 0.38 | -1.08 |
|  | Magnitude (M) | **< .001** | 2.48 ± 0.26 | 0.76 |
|  | Time (T) | **.023** | -0.54 ± 0.23 | -0.64 |
|  | B:M | .319 | -0.36 ± 0.36 | -0.99 |
|  | B:T | .375 | 0.32 ± 0.36 | 1.00 |
|  | M:T | **< .001** | -1.28 ± 0.36 | -0.99 |
|  | B:M:T | **.005** | 2.02 ± 0.72 | 1.94 |
| Theta ~ Beneficiary × Magnitude × Time | Beneficiary (B) | **.001** | -0.11 ± 0.03 | -0.08 |
|  | Magnitude (M) | .062 | -0.07 ± 0.04 | -0.12 |
|  | Time (T) | **.008** | 0.07 ± 0.02 | 0.07 |
|  | B:M | **< .001** | 0.15 ± 0.04 | 0.11 |
|  | B:T | .484 | -0.03 ± 0.04 | -0.11 |
|  | M:T | .422 | 0.03 ± 0.04 | 0.11 |
|  | B:M:T | .275 | -0.09 ± 0.08 | -0.22 |
| Delta ~ Beneficiary × Magnitude × Time | Beneficiary (B) | **< .001** | -0.27 ± 0.03 | -0.10 |
|  | Magnitude (M) | **< .001** | 0.36 ± 0.04 | 0.13 |
|  | Time (T) | .215 | -0.04 ± 0.03 | -0.09 |
|  | B:M | **< .001** | -0.22 ± 0.04 | -0.13 |
|  | B:T | **.036** | 0.09 ± 0.04 | 0.13 |
|  | M:T | .357 | -0.04 ± 0.04 | -0.13 |
|  | B:M:T | **.019** | 0.21 ± 0.09 | 0.25 |

*Notes*. Smallest effect size refers to the smallest detectable *β* at 80% power. For simplicity, only the fixed effect structure is displayed in the column detailing regression equations. Statistically significant *p* values (< .05, two-sided) are shown in bold. *SE* = standard error.

**Table S2.** Accepted ERP trials (*M* ± *SD*)

|  | Self-benefitting | Other-benefitting | *p* | Cohen’s *d* |
| --- | --- | --- | --- | --- |
| Immediate small | 38.75 ± 1.63 | 38.63 ± 1.55 | .632 | 0.08 |
| Immediate large | 37.98 ± 1.97 | 38.28 ± 2.53 | .275 | -0.18 |
| Delayed small | 38.68 ± 2.10 | 38.23 ± 2.15 | .077 | 0.29 |
| Delayed large | 38.15 ± 2.30 | 38.25 ± 1.85 | .697 | -0.06 |

*Note.* Significant differences are highlighted in bold.

**Table S3.** ANOVA results of rating data

| Source | *df* | *F* | *p* | *η_p_²* |
| --- | --- | --- | --- | --- |
| Liking |  |  |  |  |
| Beneficiary | (1, 39) | 4.08 | .050 | 0.10 |
| Magnitude | (1, 39) | 143.76 | **< .001** | 0.79 |
| Time | (1, 39) | 22.61 | **< .001** | 0.37 |
| B:M | (1, 39) | 7.50 | **.009** | 0.16 |
| B:T | (1, 39) | 9.54 | **.004** | 0.20 |
| M:T | (1, 39) | 2.73 | .107 | 0.07 |
| B:M:T | (1, 39) | 6.51 | **.015** | 0.14 |

*Note.* Significant differences are highlighted in bold.

**Table S4.** Model results of the linear mixed-effects regression for RewP and P3 data

|  | RewP | | |  | P3 | | |
| --- | --- | --- | --- | --- | --- | --- | --- |
| *Predictors* | *β* | *CI* | *p* |  | *β* | *CI* | *p* |
| (Intercept) | 6.85 | 5.63 – 8.07 | **< .001** |  | 11.77 | 10.33 – 13.22 | **< .001** |
| Beneficiary (B) | -2.51 | -3.26 – -1.76 | **< .001** |  | -2.69 | -3.45 – -1.92 | **< .001** |
| Magnitude (M) | 2.49 | 1.87 – 3.11 | **< .001** |  | 2.48 | 1.94 – 3.01 | **< .001** |
| Time (T) | -0.61 | -0.97 – -0.24 | **.001** |  | -0.54 | -1.01 – -0.08 | **.023** |
| B:M | -1.16 | -2.14 – -0.17 | **.023** |  | -0.36 | -1.06 – 0.35 | .319 |
| B:T | -0.06 | -0.78 – 0.67 | .873 |  | 0.32 | -0.39 – 1.02 | .375 |
| M:T | 0.27 | -0.46 – 0.99 | .468 |  | -1.28 | -1.98 – -0.57 | **< .001** |
| B:M:T | 1.61 | 0.16 – 3.06 | **.029** |  | 2.02 | 0.61 – 3.42 | **.005** |
| *Random Effects* |  | | |  |  | | |
| σ^2^ | 104.71 | | |  | 99.07 | | |
| τ_00_ | 14.31 _Participant_ | | |  | 20.07 _Participant_ | | |
| τ_11_ | 4.09 _beneficiary_ | | |  | 4.46 _beneficiary_ | | |
|  | 2.40 _magnitude_ | | |  | 1.50 _magnitude_ | | |
|  | 4.05 _beneficiary: magnitude_ | | |  | 0.83 _time_ | | |
| ρ_01_ | -0.42 | | |  | -0.27 | | |
|  | 0.52 | | |  | 0.37 | | |
|  | -0.31 | | |  | -0.12 | | |
| ICC | 0.13 | | |  | 0.18 | | |
| N | 40 _Participant_ | | |  | 40 _Participant_ | | |
| Observations | 12277 | | |  | 12277 | | |
| Marginal R^2^ | 0.027 | | |  | 0.029 | | |
| Conditional R^2^ | 0.157 | | |  | 0.204 | | |

*Note.* Statistics are derived from linear mixed-effects model with predictors as noted. The final model for the RewP and P3 is described using Wilkinson notation as: Amplitude ~ Beneficiary × Magnitude × Time + (Magnitude + Time + Magnitude: Time | Participant). Statistically significant *p*-values (<.05, two-sided) are shown in bold. CI = confidence interval; ICC = intraclass correlation coefficient. σ^2^ = model residuals, τ = random effects, ρ = correlation between random effects.

**Table S5.** Model results of the linear mixed-effects regression for theta and delta band data

|  | Theta | | |  | Delta | | |
| --- | --- | --- | --- | --- | --- | --- | --- |
| *Predictors* | *β* | *CI* | *p* |  | *β* | *CI* | *p* |
| (Intercept) | 0.53 | 0.42 – 0.63 | **< .001** |  | 0.85 | 0.68 – 1.02 | **< .001** |
| Beneficiary (B) | -0.11 | -0.16 – -0.05 | **.001** |  | -0.27 | -0.34 – -0.20 | **< .001** |
| Magnitude (M) | -0.07 | 0.15 – 0.00 | .062 |  | 0.36 | 0.27 – 0.45 | **< .001** |
| Time (T) | 0.07 | 0.02 – 0.12 | **.008** |  | -0.04 | -0.10 – 0.02 | .215 |
| B:M | 0.15 | 0.07 – 0.23 | **< .001** |  | -0.22 | -0.31 – -0.13 | **< .001** |
| B:T | -0.03 | -0.10 – 0.05 | .484 |  | 0.09 | 0.01 – 0.18 | **.036** |
| M:T | 0.03 | -0.05 – 0.11 | .422 |  | -0.04 | -0.13 – 0.05 | .357 |
| B:M:T | -0.09 | -0.24 – 0.07 | .275 |  | 0.21 | 0.03 – 0.39 | **.019** |
| *Random Effects* |  | | |  |  | | |
| σ^2^ | 1.19 | | |  | 1.54 | | |
| τ_00_ | 0.10 _Participant_ | | |  | 0.28 _Participant_ | | |
| τ_11_ | 0.02 _beneficiary_ | | |  | 0.03 _beneficiary_ | | |
|  | 0.04 _magnitude_ | | |  | 0.05 _magnitude_ | | |
|  | 0.01 _time_ | | |  | 0.02 _time_ | | |
| ρ_01_ | -0.30 | | |  | -0.60 | | |
|  | -0.59 | | |  | 0.66 | | |
|  | -0.00 | | |  | 0.16 | | |
| ICC | 0.09 | | |  | 0.16 | | |
| N | 40 _Participant_ | | |  | 40 _Participant_ | | |
| Observations | 12277 | | |  | 12277 | | |
| Marginal R^2^ | 0.005 | | |  | 0.029 | | |
| Conditional R^2^ | 0.098 | | |  | 0.188 | | |

*Note.* Statistics are derived from linear mixed-effects model with predictors as noted. The final model for the theta and delta is described using Wilkinson notation as: Power ~ Beneficiary × Magnitude × Time + (Beneficiary + Magnitude + Time| Participant). “:” indicates interactions between predictors. Statistically significant *p*-values (<.05, two-sided) are shown in bold. CI = confidence interval; ICC = intraclass correlation coefficient. σ^2^ = model residuals, τ = random effects, ρ = correlation between random effects.
